# Maternal pre-pregnancy body mass index, gestational weight gain and breastfeeding outcomes: a cross-sectional analysis

**DOI:** 10.1186/s12884-020-03156-8

**Published:** 2020-08-17

**Authors:** Hayley Martin, Kelly Thevenet-Morrison, Ann Dozier

**Affiliations:** Public Health Sciences, University of Rochester School of Medicine and Dentistry, 265 Crittenden Blvd. CU 420644, Rochester, NY 14642 USA

**Keywords:** Pregnancy weight gain, Pre-pregnancy BMI, Pregnancy, Lactation, Exclusive breastfeeding, Breastfeeding duration, Breastfeeding cessation, Infant feeding

## Abstract

**Background:**

It is well established that mothers with above-normal pre-pregnancy BMI are at increased risk of breastfeeding cessation; however, the impact of pregnancy weight-gain (PWG) is less well-defined. Excess PWG may alter the hormonal preparation of breast tissue for lactation, increase the risk of complications that negatively impact breastfeeding (e.g. Cesarean-section, gestational diabetes), and may make effective latch more difficult to achieve.

**Methods:**

Our objective was to determine the impact of PWG and pre-pregnancy BMI on the risk of breastfeeding cessation utilizing the Institute of Medicine’s 2009 recommendations. Cox proportional hazards models were utilized to estimate the risk of cessation of exclusive breastfeeding, and cessation of any breastfeeding among women who initiated exclusive and any breastfeeding, respectively, in a cross sectional sample of survey respondents from a New York county (*N* = 1207). PWG category was interacted with pre-pregnancy BMI (3 levels of pre-pregnancy BMI, 3 levels of PWG). Confounders of the relationship of interest were evaluated using directed acyclic graphs and bivariate analyses; variables not on the proposed causal pathway and associated with the exposure and outcome were included in multivariate models. Results: After adjustment, women of normal and obese pre-pregnancy BMI with greater-than-recommended PWG had 1.39 (1.03–1.86) and 1.48 (1.06–2.07) times the risk of any breastfeeding cessation within the first 3 months postpartum compared to women with normal pre-pregnancy BMI who gained within PWG recommendations. Overweight women with greater-than-recommended PWG were at increased risk of cessation, although not significantly (aHR [95% CI]: 1.29 [0.95–1.75]). No significant relationship was observed for exclusive breastfeeding cessation.

**Conclusions:**

Pre-pregnancy BMI and PWG may be modifiable risk factors for early breastfeeding cessation. Understanding the mechanism behind this risk should be ascertained by additional studies aimed at understanding the physiological, social, logistical (positioning) and other issues that may lead to early breastfeeding cessation.

## Background

As overweight and obesity continue to rise among women of reproductive age [1], a comprehensive understanding of the interplay between maternal adiposity and breastfeeding outcomes is needed. While a negative relationship between increasing pre-pregnancy body mass index (BMI) and breastfeeding outcomes has been demonstrated in numerous studies, these studies often do not account for the amount of weight gained over the course of pregnancy. Additionally, mothers who begin their pregnancy with a higher than normal BMI (≥ 25) are at increased risk of gaining beyond the currently recommended weight during pregnancy [2].

The Institute of Medicine’s^1^ recommendations for maternal weight gain during pregnancy were last updated in 2009 [3], and differ from previous guidelines by providing a specific, limited range of recommended weight gain for women with obesity (BMI ≥ 30 kg/m^2^), where the previous recommendations were limited to women with a BMI of 29 kg/m^2^ or less [4]. The updated guidelines are aimed at preventing of poor birth outcomes including abnormal birthweight and fetal growth, as well as postpartum maternal weight retention [5]. These guidelines are listed in Table 1.

**Table 1:** Institute of Medicine 2009 total pregnancy weight gain guidelines

Pre-pregnancy BMI Category	Recommended Total Weight Gain during pregnancy (kg)
Underweight < 18.5 kg/m^2^	12.5–18.0
Normal Weight 18.5–24.9 kg/m^2^	11.5–16
Overweight 25.0–29.9 kg/m^2^	7.0–11.5
Obese ≥ 30.0 kg/m^2^	5.0–9.0

Adapted from Institute of Medicine, 2009 [5]

The interplay between pre-pregnancy BMI and pregnancy weight gain, and their impact on breastfeeding outcomes is likely a combination of effects mediated through physiological differences, as well as psychosocial and health behavior factors [6, 7]. Physiological alterations due to weight status both pre-pregnancy and during gestation may influence lactation via changes in mammary tissue preparation, progression of lactation (e.g. delayed transition from stage I to stage II lactogenesis), as well as pregnancy and infant complications that impact breastfeeding [8, 9].

Given the nutritional importance of breastfeeding and the health benefits imparted to both mother and child [10–12], a clear understanding of the impact of weight gain during pregnancy is needed in the context of the current pregnancy weight gain guidelines. Furthermore, evaluating the potential modification of this relationship by pre-pregnancy BMI is important. There is a well-established link between high pre-pregnancy BMI and poor breastfeeding outcomes, and previous studies demonstrated a significant interaction effect with pregnancy weight gain and baseline maternal BMI utilizing categories based on the Institute of Medicine’s 1990 pregnancy weight gain guidelines [13, 14]. An updated assessment of this relationship is needed for public health messaging and clinical guidance for mothers at risk of poor breastfeeding outcomes in the United States related to these factors. This study aims to evaluate the impact of pregnancy weight gain on the duration of any and exclusive breastfeeding among breastfeeding initiators, in the context of pre-pregnancy BMI. We hypothesized that greater than recommended maternal weight gain would result in increased risk of breastfeeding cessation in all pre-pregnancy BMI categories.

## Methods

This is a secondary analysis using a cross sectional data set collected by Dozier and colleagues in order to evaluate breastfeeding outcomes. The data were obtained from two linked sources; a single-time point mailed survey based on the Pregnancy Risk Assessment Monitoring System methodology (PRAMS) using PRAMS version 6.0 items that contained questions regarding maternal beliefs, behaviors and experiences during the pre-pregnancy through postpartum period (data source one) [15]. As described in Dozier et al. [16], a random sample of all mothers residing in Monroe County, New York with a live birth were mailed the survey instrument at approximately 4 months postpartum to women who had given birth between May 2009 and April 2012. Randomization was carried out using (a random number generator in SAS) and stratifying based on income status using a birth certificate registry that includes all hospital births in the county (representing 99% of all births). Those with Medicaid funded birth and/or prenatal WIC enrollment were considered to have lower-income status and were oversampled based on an anticipated 35% response rate (the expected response rate for those categorized as non-low income was 55%) in order to obtain a distribution of income status representative of the population in the final sample. The survey responses were then matched with the birth certificate (data source two; US Standard Certificate of Live Birth; 11/2003 revision, which contained additional information including demographics, medical information and history, delivery characteristics and birth hospitalization information for both mother and baby.

Birth certificate data were abstracted after delivery by trained birth certificate registrars based on the mothers’ prenatal and delivery records and infants’ medical records. This process is a standard part of state-mandated reporting for all live births, and is completely independent from the study protocol.

Inclusion criteria for this secondary analysis were: initiation of breastfeeding, complete responses to infant feeding questions allowing for determination of breastfeeding initiation, duration and exclusivity, complete height and pre-pregnancy weight for calculation of pre-pregnancy BMI category, non-underweight pre-pregnancy BMI, data on total weight gain during pregnancy, and reporting that the infant was alive at the time of survey completion. The latter criteria would not contribute to selection bias as infant mortality in this community is < 7 per 1000 births.

Breastfeeding initiation (any and exclusive) was defined using data from the birth certificate in combination with survey responses. The birth certificate indicates whether mothers during the delivery hospitalization initiated exclusive or non-exclusive (i.e. was formula administered in the hospital along with breastfeeding) or did not initiate breastfeeding. The timing of breastfeeding initiation is assumed to be within the first 24 h of life. In the rare cases where mothers initiated formula feeding only during the hospital and subsequently began breast feeding after they had been discharged from the hospital, their duration of any breastfeeding reflected this difference in our analyses. Survey responses were used to categorize the duration of any breastfeeding among all women who initiated breastfeeding, and the duration of exclusive breastfeeding among initiators of exclusive breastfeeding. Exclusive breastfeeding categorization considered not only the addition of formula, but also complementary foods and liquids. A mother’s duration of exclusive breastfeeding was categorized as the number of days of providing her baby only human milk, without the use of infant formula or other foods or liquids. The duration of any breastfeeding was categorized as the number of days a mother provided human milk to their baby, regardless of other sources of nutrition. Mothers who were still providing human milk (exclusively, or in addition to other foods) at the time of survey completion were censored at 3 months for these analyses.

Pre-pregnancy BMI was categorized into underweight (excluded), normal, overweight and obese based on the categories listed in Table 1, using the height and pre-pregnancy weight data abstracted by trained hospital birth registrars from the mothers’ prenatal records and entered into the corresponding fields of the electronic birth certificate. The final pregnancy weight was similarly abstracted from the hospital/labor medical record for entry into the birth certificate. This data source was utilized for weight and height, to minimize social desirability bias commonly associated with self-reported data.

Pre-pregnancy weight is what is documented on the prenatal record by the provider (and subsequently entered into the birth certificated (by the hospital birth registrars). This may be based on weight at the first prenatal visit or at the most recent pre-pregnancy visit. The weight at the end of pregnancy is abstracted from the documentation at the time of the mother’s admission when in labor. This is most commonly an actual weight. If that is not feasible then the last prenatal weight may be used or the field is left blank.

Pregnancy weight gain was categorized based on the Institute of Medicine’s 2009 recommendations was determined by subtracting pre-pregnancy weight from final pregnancy weight of each mother. An interaction term including these two variables was created to allow investigation of the impact of pregnancy weight gain at each level of pre-pregnancy BMI. All combinations of categories of pregnancy weight gain and pre-pregnancy BMI were compared to women who gained as recommended and had a normal pre-pregnancy BMI; this served as the reference group for all other comparisons. Women who were recorded as having an underweight pre-pregnancy BMI were excluded given that the number of women in this category was small, and precluded their inclusion in statistical modeling.

Additional covariates considered in this analysis included: maternal age (continuous) maternal education (less than Bachelor’s degree/Bachelor’s degree or higher), race and ethnicity (white non-Hispanic/other), income status (low and non-low income; low income defined as enrollment in prenatal WIC and/or Medicaid funded delivery), marital status (married/not married), parity (continuous), smoking in previous 2 years (yes/no), infant sex (female/male) and vaginal delivery (yes/no). Education and race and ethnicity categories are expanded in Table 1 for descriptive purposes, but these variables were operationalized as above for all statistical analyses to maximize statistical efficiency. All covariates evaluated utilized data from the birth certificate. To categorize income status and smoking in the last 2 years, responses from the survey were used when birth certificate information was missing.

Descriptive statistics (mean and standard deviation for continuous variables, number and percent for categorical variables) were used to characterize the sample. Bivariate associations of each outcome (time to any and exclusive breastfeeding cessation) and each covariate were evaluated using univariate Cox proportional hazard models. Bivariate associations between the exposure (pregnancy weight gain category) and each covariate were evaluated using Chi square tests (categorical), ANOVA (continuous, normal) or Kruskal-Wallis test (continuous, non-normal). Directed acyclic graphs were used to evaluate the potential confounding structure of the relationships of interest. Covariates were considered confounders of the association of interest if they were associated with both the outcome and exposure variables, and not on the proposed causal pathway.

Confounders were included in adjusted Cox-Proportional Hazards models of cessation of any breastfeeding among all women who initiated breastfeeding, and cessation of exclusive breastfeeding among only those mothers who initiated exclusive breastfeeding. For both models, pregnancy weight gain was the exposure of interest, and this term was interacted with pre-pregnancy BMI. Mothers with missing values on included confounders were excluded from these analyses. Unfortunately, due to a small number of mothers in the underweight BMI category, it was necessary to exclude these subjects from analyses. Continuous categorizations of BMI and pregnancy weight gain were not utilized given evidence of a non-linear relationship with the outcome of interest. Cox-proportional hazards model assumptions were checked using graphical assessment as well as interacting each covariate with time (PHREG procedure, SAS). SAS software version 9.4 and R version 1.1.453 were used for analysis [17, 18].

## Results

**Fig. 1:**
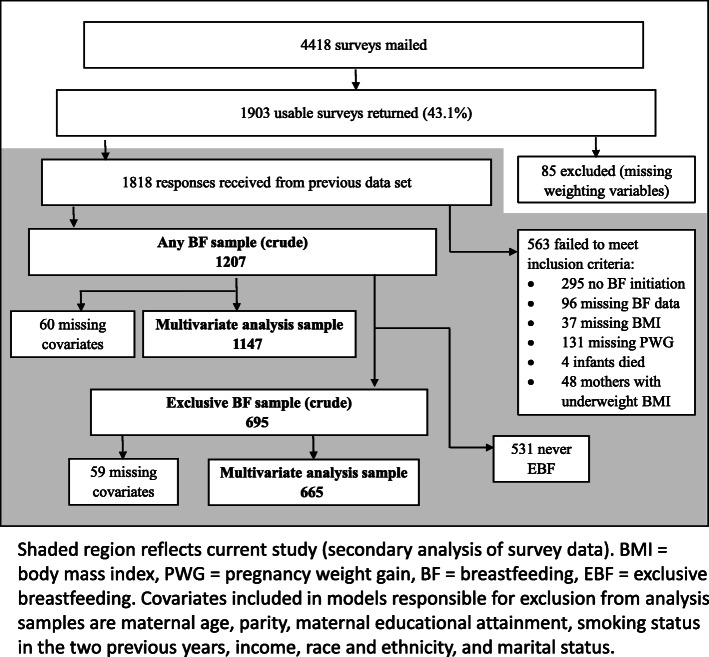
Survey administration, response, and inclusion in current analysis flow chart

A total of 4418 surveys were mailed successfully, and 1903 surveys were returned by respondents (43.1% response rate). Eighty-five mothers were excluded due to missing data required for survey weighting for the original analysis. Women were excluded for not meeting the following criteria: did not initiate breastfeeding (N excluded = 295), incomplete responses to infant feeding questions allowing for determination of breastfeeding duration and exclusivity (N excluded = 96), incomplete height and pre-pregnancy weight for calculation of pre-pregnancy BMI category (N excluded = 37), and missing data on total weight gain during pregnancy (N excluded = 131). Four women’s responses were excluded because the baby was not alive at the time of survey completion. Forty-eight mothers were excluded who had below normal (18.5 kg/m^2^) pre-pregnancy BMI. The final study sample was made up of 1207 respondents (Fig. 1).

Over half of women gained more weight and 18% gained less weight during pregnancy than was recommended based on their pre-pregnancy BMI (per Institute of Medicine guidelines). Nearly half of women had a pre-pregnancy BMI in the overweight or obese range (> 24.9 kg/m^2^). 57.6% of the sample initiated exclusive breastfeeding during the birth hospitalization. Overall, the median duration of any breastfeeding in the sample was 106 days (interquartile range 42–123), and the median duration of exclusive breastfeeding (among those who initiated exclusive breastfeeding) was 90 days (interquartile range 30–117). By 3 months postpartum, 40.7% of mothers who had initiated any breastfeeding had stopped breastfeeding, and 42.6% of mothers who had initiated exclusive breastfeeding were no longer exclusively breastfeeding their child. Most women had at least some college education, identified as white, non-Hispanic, and were married, while nearly half of the sample were from low-income households (Table 2).

**Table 2:** Sample characteristics by Pregnancy Weight Gain (PWG) Category

	Total Sample	Within recommendations	Less than recommended	Greater than recommended	
	*N* = 1207	*N* = 372	*N* = 211	*N* = 624	*P*-value
**Breastfeeding initiation**	N (% of total sample)	N (% of respective PWG Category)			
Exclusive breastfeeding	695 (57.6)	227 (61.0)	111 (52.6)	357 (57.2)	0.137
Combination feeding^a^	512 (42.4)	145 (39.0)	100 (47.4)	267 (42.8)	
	Median (Interquartile Range)				
**Exclusive Breastfeeding Duration (days)**^b^	90 (30–117)	98 (42–120)	90 (28–120)	75 (28–109)	0.004^c^
**Any Breastfeeding Duration (days)**	106 (42–123)	110 (60–124)	106 (42–123)	101 (42–123)	0.002^c^
**Pre-pregnancy BMI Category**	N (% of total sample)	N (% of respective PWG Category)			
Normal (18.5–24.9 kg/m2)	623 (51.6)	266 (71.5)	111 (52.6)	246 (39.4)	< 0.001
Overweight (25.0–29.9 kg/m2)	289 (23.9)	45 (12.1)	35 (16.6)	209 (33.5)	
Obese (30.0 or higher kg/m2)	295 (24.4)	61 (16.4)	65 (30.8)	169 (27.1)	
	Mean (sd)				
**Maternal Age (years)**	29.25 (5.70)	30.00 (5.44)	29.19 (5.89)	28.82 (5.75)	0.007^d^
**Maternal Education**	N (% of total sample)	N (% of respective PWG Category)			
Bachelor’s or more	548 (45.4)	214 (57.5)	83 (39.3)	251 (40.2)	< 0.001^e^
Some college	313 (25.9)	75 (20.2)	53 (25.1)	185 (29.6)	
High school or less	345 (28.6)	83 (22.3)	74 (35.1)	188 (30.1)	
Missing^f^	1 (0.1)	0 (0.0)	1 (0.5)	0 (0.0)	
**Race and Ethnicity**					
White non-Hispanic	856 (70.9)	290 (78.0)	123 (58.3)	443 (71.0)	< 0.001^e^
Black non-Hispanic	182 (15.1)	39 (10.5)	47 (22.3)	96 (15.4)	
Other non-Hispanic	49 (4.1)	20 (5.4)	13 (6.2)	16 (2.6)	
Hispanic	66 (5.5)	12 (3.2)	16 (7.6)	38 (6.1)	
Missing ^f^	54 (4.5)	11 (3.0)	12 (5.7)	31 (5.0)	
**Income status**^g^					
Low Income	521 (43.2)	124 (33.3)	108 (51.2)	289 (46.3)	< 0.001
**Marital status**					
Married	785 (65.0)	273 (73.4)	127 (60.2)	385 (61.7)	< 0.001
	Mean (sd)				
**Parity**	0.84 (1.14)	0.82 (1.04)	1.14 (1.39)	0.75 (1.09)	< 0.001^d^
N Missing (%) ^f^	6 (0.5)	1 (0.3)	0 (0.0)	5 (0.8)	
**Smoking (last 2 years)**					
Yes	310 (25.7)	82 (22.0)	45 (21.3)	183 (29.3)	0.011
**Infant sex**					
Female	577 (47.8)	190 (51.1)	99 (46.9)	288 (46.2)	0.310
**Vaginal delivery**					
Yes	858 (71.1)	271 (72.8)	158 (74.9)	429 (68.8)	0.157

^a^Human milk and formula ^b^Among those who initiated EBF ^c^*p* value derived from Kruskal-Wallis test ^d^*p* value derived from ANOVA test ^e^These categories were collapsed into dichotomous variables (Bachelor’s or more versus less than Bachelor’s degree and white non-Hispanic versus other, respectively) for multivariate modeling in order to maximize statistical efficiency, and are reported here in detail for descriptive purposes. *P*-values reported are from chi-square tests utilizing the dichotomous categorization of these variables. ^f^Missing values not included in any statistical tests ^g^Prenatal WIC enrollment and/or Medicaid funded delivery

All subjects in study sample had complete data for variables without a “missing” category, all *p* values from Chi square test unless otherwise noted

Breastfeeding duration (any and exclusive) was significantly associated with pregnancy weight gain in bivariate analysis. Women who gained within the Institute of Medicine’s 2009 recommendations on average, were older, had more education, higher income, and were more often married than women who gained outside of recommendations. Race and ethnicity, parity and smoking status in the 2 years prior to the survey were also significantly associated with maternal weight gain during pregnancy (Table 2). Both time to cessation of any and exclusive breastfeeding and pregnancy weight gain category were associated with maternal age, parity, maternal educational attainment, smoking status in the two previous years, income status, race and ethnicity, and marital status. These variables were considered confounders and included in each multivariable model. Supplemental Tables 1 and 2 contain crude and adjusted effect estimates, respectively, for all variables included in each model.

**Fig. 2:**
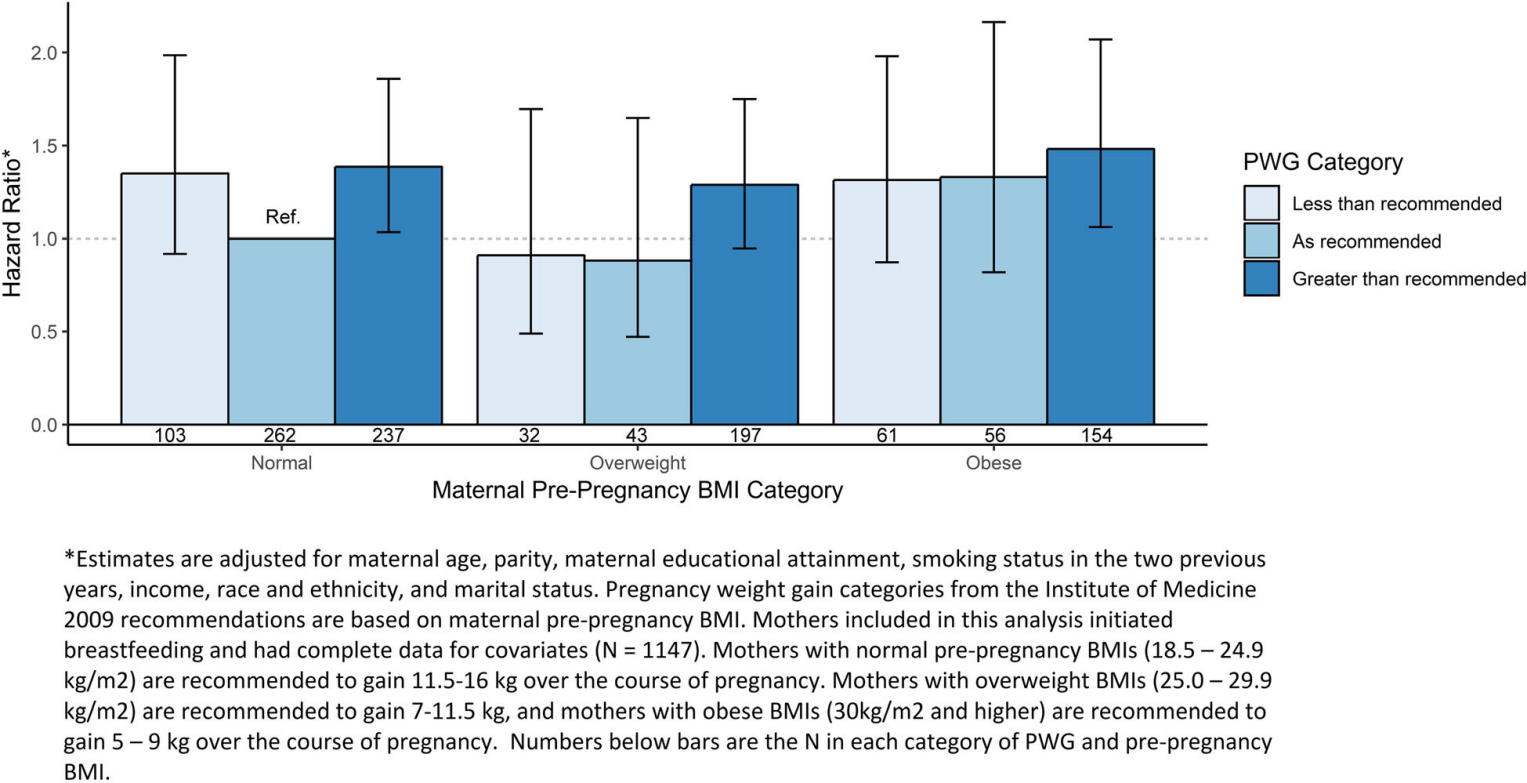
Adjusted Relative Hazard of Any Breastfeeding Cessation by Pre-Pregnancy BMI category and Pregnancy Weight Gain (PWG) Category from Birth through 3 Months Postpartum

After adjustment for confounders, women of normal and obese pre-pregnancy BMI who gained in excess of the Institute of Medicine guidelines were at significantly increased risk of breastfeeding cessation by 3 months compared to the reference group of women with normal BMI who gained as recommended (aHR [95% CI]: 1.39 [1.03–1.86] and 1.48 [1.06–2.07], respectively). Overweight women who gained more weight than recommended were at increased risk of cessation, although not significantly (aHR [95% CI]: 1.29 [0.95–1.75]). Women with pre-pregnancy obesity showed a trend toward increased risk cessation in both less than recommended and as recommended PWG categories (aHR [95% CI]: 1.32 [0.87–1.98] and 1.33 [0.82–2.16], respectively). Women of normal pre-pregnancy BMI who gained less than recommended showed a trend towards increased risk of cessation compared to the reference group (aHR [95%CI]: 1.35 [0.92–1.99]) (Fig. 2).

**Fig. 3:**
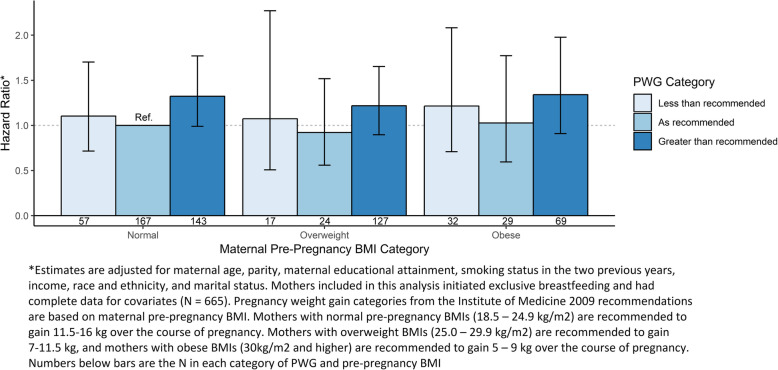
Adjusted Relative Hazard of Exclusive Breastfeeding Cessation by Pre-Pregnancy BMI category and Pregnancy Weight Gain (PWG) Category from Birth through 3 Months Postpartum

A similar trend was observed for the risk of cessation of exclusive breastfeeding through 3 months among the subset of women who initiated exclusive breastfeeding. After controlling for confounders, a trend towards increased risk of breastfeeding cessation was seen among each pre-pregnancy BMI category in women who gained in excess of the 2009 Institute of Medicine recommendations compared to women with normal pre-pregnancy BMI who gained within recommendations (aHR [95%CI]: normal BMI 1.32 [0.99–1.77]; overweight BMI 1.21 [0.90–1.65]; obese BMI 1.34 [0.91–1.98]) (Fig. 3). Women who gained less than recommended showed a smaller, non-significant increase in exclusivity cessation risk (aHR [95%CI]: normal BMI 1.10 [0.72–1.70]; overweight BMI 1.07 [0.51–2.27]; obese BMI 1.21 [0.71–2.08]). Women who gained as recommended in the overweight and obese categories did not see an increased risk of cessation of exclusivity compared to the reference group (aHR [95%CI] 0.92 [0.56–1.52] and 1.02 [0.60–1.77], respectively).

## Discussion

The risk of cessation of breastfeeding was increased among women who gained excess weight during pregnancy for women of normal and obese pre-pregnancy BMI, and a similar, although non-significant trend was observed for women with overweight pre-pregnancy BMI. The association between these variables and time to exclusive breastfeeding cessation was attenuated in comparison. Overall, women who gained within the Institute of Medicine’s guidelines tended to demonstrate lower risk of breastfeeding cessation compared to women who gained outside of these recommendations in their pre-pregnancy BMI category.

These findings are similar to results from previous literature [13, 14] utilizing the Institute of Medicine’s 1990 guidelines; however, this is the first study that has found a significant effect of pregnancy weight gain since the 2009 guidelines were released. A study conducted in a US cohort, found no significant impact of pregnancy weight gain after adjustment for confounders [19]. Two cohort studies evaluating the impact of pregnancy weight gain and pre-pregnancy BMI on breastfeeding outcomes conducted in Brazil and China [20, 21]. The Brazilian study found no differences in any or exclusive breastfeeding duration by pregnancy weight gain category, and did not evaluate the interaction between pregnancy weight gain and pre-pregnancy BMI. The study conducted in China considered the interaction between these two factors, and similarly found no differences by pregnancy weight gain category. Importantly, this study’s BMI and pregnancy weight gain categorization was based on the Chinese classification system, which differs slightly from the Institute of Medicine’s recommendations. Differences in sample size as well as regional and global breastfeeding behavior, norms, and predictors may explain the differences in results observed in the current study.

The implications of these findings are an important factor for maternal and infant health extending well beyond the early postpartum period. Mothers with increased pre-pregnancy BMI and excess gestational weight gain are at increased risk of diabetes mellitus, higher BMI in later pregnancies, and greater risk of depression [22, 23]. Breastfeeding reduces the risk of diabetes [24–28], is associated with less postpartum weight retention [29–36], and may be a protective factor for depression in the postpartum period [37–42]. Women with above normal BMI also have increased risk of postmenopausal breast cancer [43–45] and ovarian cancer [45], both of which are negatively associated with breastfeeding [46]. These associations highlight the important risk reduction that mothers at increased risk due to high BMI and excess weight gain could experience with improved breastfeeding outcomes.

Given the numerous maternal health benefits imparted by breastfeeding, especially given the increased risk state of mothers with above normal BMI or excess pregnancy weight gain, efforts to increase the proportion of infants who receive optimal nutrition in the first months of life is warranted, and should include information about appropriate weight gain during pregnancy. For mothers who prenatally intend to breastfeed, it may be helpful to provide them with information about improved breastfeeding duration among mothers who gain within the Institute of Medicine’s recommendations. For mothers who gain above recommendations, anticipatory guidance regarding common breastfeeding problems, additional breastfeeding support especially in the prenatal and early postpartum periods, and general education about the benefits of breastfeeding for mom and baby tailored to the risk factors most relevant to the individual mother may be helpful to improve breastfeeding outcomes. Individuals providing breastfeeding support should consider weight gain above the Institute of Medicine recommendations as a potential risk factor and increase the frequency of contact, assessment, and interventions, particularly in the hospital and immediate postpartum period.

Additional factors that could not be evaluated in this analysis include gestational diabetes, hypertensive disorders of pregnancy, postpartum hemorrhage, pre-eclampsia and C-section. These conditions are more common among women who gain excess weight during pregnancy and increase the risk of maternal intensive care unit admission and longer hospital stays, however, given that this increased risk is conferred at least partially *from* this excess weight gain, placing these important predictors of breastfeeding outcomes on the causal pathway between pregnancy weight gain and the outcomes evaluated. Including these conditions as covariates in multivariable models of breastfeeding outcomes would have resulted in underestimation of the association of interest. Due to a limited total sample size and low occurrence rates for these pregnancy complications in our sample it was not possible to evaluate these factors as potential mediators of the association between pregnancy weight gain and breastfeeding outcomes. Given the biological importance of these factors for successful lactation outside of maternal decision making (establishment of milk supply, timing of mature milk production, etc), future studies in large cohorts should evaluate whether a portion of the increased risk of poor breastfeeding outcomes among mothers who gain more than is recommended is due to these pregnancy and birth factors. Due to the small number of women with underweight pre-pregnancy BMIs, we were unable to evaluate the impact of pregnancy weight gain and weight status on breastfeeding outcomes in these women. Additionally, self-reported breastfeeding data were utilized for this study. As noted, the source of weight data on the birth certificate is consistent (prenatal/hospital records), however it is possible the some of the weights in these records were drawn from prior visit documentation rather than direct measurement.

While studies have demonstrated that PRAMS self-report measures of breastfeeding initiation show a high degree of agreement with the birth certificate [47], similar studies evaluating post-hospital breastfeeding outcomes are not available. We estimate that on average, breastfeeding duration may have been mildly overestimated due to social desirability bias, however, we do not believe this would be differential with respect to the exposure of interest, and therefore feel any bias in the effect estimates reported is likely in the direction of the null hypothesis. Additionally, given that that breastfeeding data for women who chose not respond to the survey, it is unclear if this sample is truly representative of the community, and is likely that responders were more likely to be breastfeeders. Importantly, the relationship between pregnancy weight gain and breastfeeding outcomes may be different between survey responders and non-responders. Utilizing data from sources less prone to response bias, social desirability and recall biases (e.g. the child’s pediatric record) would be a valuable addition to future studies.

Despite these limitations, this analysis has several key strengths. The sample of respondents included in this study are representative of the county from which they were sampled allowing for evaluation of this relationship in a sample that represents the communities with similar demographic characteristics. These results are likely generalizable to populations with similar characteristics, however, are not generalizable to women with underweight pre-pregnancy BMIs and should be replicated in other populations. Categorizing pre-pregnancy BMI status and pregnancy weight gain using birth certificate data (originally abstracted directly from the medical record by birth certificate coders) eliminates concerns about social desirability bias that is often present in self-report data regarding weight. Additionally, this association should be evaluated in regions with greater diversity in terms of cultural, ethnic and racial backgrounds, and with differing health care infrastructure around pregnancy and breastfeeding support in order to understand the importance of these factors in the association of interest. Finally, having data regarding breastfeeding outcomes extending to 4 months allowed us to evaluate the risk of cessation over a long follow up period, and provides detailed time to event breastfeeding data that captures the individual variation in breastfeeding outcomes in this population.

## Conclusions

Weight gain in excess of the Institute of Medicine’s 2009 guidelines leads to increased risk of poor breastfeeding outcomes. Efforts to reduce overweight and obesity in women before child bearing, in addition to increasing the number of mothers who gain weight during pregnancy within Institute of Medicine guidelines would be expected to improve breastfeeding outcomes. Given that these modifiable breastfeeding risk factors impact an increasingly large proportion of pregnant mothers in the United States, effective interventions aimed at not only lowering pre-pregnancy BMI, but also reducing excessive weight gain during pregnancy could significantly improve breastfeeding outcomes on a national scale.

## Supplementary information

**Additional file 1: Supplemental Table 1.** Crude: Time to cessation of breastfeeding ~ Pregnancy weight gain (PWG)* BMI.

**Additional file 2: Supplemental Table 2.** Adjusted: Time to cessation of breastfeeding ~ Pregnancy weight gain (PWG)*BMI.

## Data Availability

The datasets used and/or analyzed during the current study are available from the corresponding author on reasonable request.

## Abbreviations

aHR: Adjusted Hazard Ratio
ANOVA: Analysis of variance
BMI: Body Mass Index
CI: Confidence Interval
PRAMS: Pregnancy Risk Assessment Monitoring System
PWG: Pregnancy Weight Gain

## Footnotes

1 renamed National Academy of Medicine in 2015, but will be referred to by the name of the published guidelines throughout this text
