# Supplementary material for: Maternal pre-pregnancy body mass index, gestational weight gain and breastfeeding outcomes: a cross-sectional analysis
Source: BMC Pregnancy Childbirth. 2020 Aug 17;20:471. doi: 10.1186/s12884-020-03156-8 (PMC7433137; doi:10.1186/s12884-020-03156-8)
Supplement: Supplementary file 1 — Additional file 1: Supplemental Table 1. Crude: Time to cessation of breastfeeding ~ Pregnancy weight gain (PWG)* BMI. [file 12884_2020_3156_MOESM1_ESM.docx]

| Supplemental Table 1. Crude: Time to cessation of breastfeeding ~ Pregnancy weight gain (PWG)* BMI | | | | | | |
| --- | --- | --- | --- | --- | --- | --- |
|  | Any Breastfeeding | | | Exclusive breastfeeding | | |
| Parameter | Hazard Ratio | 95% Hazard Ratio Confidence Limits | | Hazard Ratio | 95% Hazard Ratio Confidence Limits | |
| Normal BMI Recommended PWG | 1.00 | (Ref) | | 1.00 | (Ref) | |
| Normal BMI Less than rec PWG | 1.424 | 0.986 | 2.057 | 1.214 | 0.801 | 1.841 |
| Normal BMI More than rec PWG | 1.583 | 1.187 | 2.111 | 1.540 | 1.155 | 2.054 |
| Overweight BMI Recommended PWG | 0.973 | 0.551 | 1.719 | 1.099 | 0.667 | 1.812 |
| Overweight BMI Less than rec PWG | 1.233 | 0.674 | 2.256 | 1.432 | 0.713 | 2.877 |
| Overweight BMI More than rec PWG | 1.628 | 1.207 | 2.197 | 1.512 | 1.119 | 2.042 |
| Obese BMI Recommended PWG | 1.847 | 1.194 | 2.855 | 1.464 | 0.897 | 2.389 |
| Obese BMI Less than rec PWG | 1.905 | 1.283 | 2.831 | 1.582 | 0.997 | 2.512 |
| Obese BMI More than rec PWG | 2.010 | 1.480 | 2.729 | 1.764 | 1.241 | 2.508 |

Supplemental Table 1. Unadjusted Cox Proportional Hazards Models were used to estimate the hazard ratio of any and exclusive breastfeeding cessation by pre-pregnancy BMI category and pregnancy weight gain (PWG) category from delivery through 3 months postpartum. Mothers included in these models initiated any (N = 1207; 59.3% censored) or exclusive (N = 695; 42.6% censored) breastfeeding, respectively. Pregnancy weight gain categories from the Institute of Medicine 2009 recommendations are based on maternal pre-pregnancy BMI. Mothers with normal pre-pregnancy BMIs (18.5 – 24.9 kg/m2) are recommended to gain 11.5-16 kg over the course of pregnancy. Mothers with overweight BMIs (25.0 – 29.9 kg/m2) are recommended to gain 7-11.5 kg, and mothers with obese BMIs (30kg/m2 and higher) are recommended to gain 5 – 9 kg over the course of pregnancy.
