# Supplementary material for: Maternal pre-pregnancy body mass index, gestational weight gain and breastfeeding outcomes: a cross-sectional analysis
Source: BMC Pregnancy Childbirth. 2020 Aug 17;20:471. doi: 10.1186/s12884-020-03156-8 (PMC7433137; doi:10.1186/s12884-020-03156-8)
Supplement: Supplementary file 2 — Additional file 2: Supplemental Table 2. Adjusted: Time to cessation of breastfeeding ~ Pregnancy weight gain (PWG)*BMI. [file 12884_2020_3156_MOESM2_ESM.docx]

| Supplemental Table 2. Adjusted: Time to cessation of breastfeeding ~ Pregnancy weight gain (PWG)*BMI | | | | | | |
| --- | --- | --- | --- | --- | --- | --- |
|  | Any Breastfeeding | | | Exclusive breastfeeding | | |
| Parameter | Hazard Ratio | 95% Hazard Ratio Confidence Limits | | Hazard Ratio | 95% Hazard Ratio Confidence Limits | |
| Normal BMI Recommended PWG | 1.00 | (Ref) | | 1.00 | (Ref) | |
| Normal BMI Less than rec PWG | 1.350 | 0.918 | 1.985 | 1.104 | 0.716 | 1.702 |
| Normal BMI More than rec PWG | 1.386 | 1.033 | 1.858 | 1.323 | 0.990 | 1.770 |
| Overweight BMI Recommended PWG | 0.882 | 0.471 | 1.649 | 0.921 | 0.559 | 1.518 |
| Overweight BMI Less than rec PWG | 0.911 | 0.489 | 1.697 | 1.074 | 0.508 | 2.271 |
| Overweight BMI More than rec PWG | 1.289 | 0.948 | 1.751 | 1.218 | 0.897 | 1.653 |
| Obese BMI Recommended PWG | 1.331 | 0.819 | 2.164 | 1.027 | 0.595 | 1.773 |
| Obese BMI Less than rec PWG | 1.315 | 0.873 | 1.980 | 1.215 | 0.709 | 2.082 |
| Obese BMI More than rec PWG | 1.482 | 1.061 | 2.070 | 1.341 | 0.910 | 1.977 |
| Maternal age (continuous) | 0.972 | 0.952 | 0.993 | 1.000 | 0.976 | 1.024 |
| Parity (continuous) | 1.009 | 0.925 | 1.102 | 0.903 | 0.814 | 1.002 |
| Maternal Education: Bachelor’s or higher vs less than Bachelor’s | 0.551 | 0.429 | 0.708 | 0.611 | 0.473 | 0.790 |
| Smoking in the previous 2 years: Yes vs no | 1.457 | 1.187 | 1.789 | 1.397 | 1.095 | 1.783 |
| Low income vs non-Low income | 0.966 | 0.757 | 1.231 | 0.870 | 0.663 | 1.142 |
| Other race/ethnicity vs white non-Hispanic | 1.032 | 0.824 | 1.292 | 1.403 | 1.084 | 1.815 |
| Married vs not married | 0.718 | 0.567 | 0.908 | 0.957 | 0.738 | 1.243 |

Supplemental Table 2. Multivariable Cox Proportional Hazards Models were used to estimate the adjusted hazard ratio of any and exclusive breastfeeding cessation by pre-pregnancy BMI category and pregnancy weight gain (PWG) category from delivery through 3 months postpartum. Mothers included in these models initiated any (N = 1147; 60.9% censored) or exclusive (N = 665; 43.3% censored) breastfeeding, respectively and were not missing values for the following covariates also included in these models: maternal age, parity, maternal education, smoking in the past two years, income status, race and ethnicity, and marital status. Reference values for categorical covariates are the second listed (eg. Less than Bachelor’s degree is the reference value for the maternal education variable). Pregnancy weight gain categories from the Institute of Medicine 2009 recommendations are based on maternal pre-pregnancy BMI. Mothers with normal pre-pregnancy BMIs (18.5 – 24.9 kg/m2) are recommended to gain 11.5-16 kg over the course of pregnancy. Mothers with overweight BMIs (25.0 – 29.9 kg/m2) are recommended to gain 7-11.5 kg, and mothers with obese BMIs (30kg/m2 and higher) are recommended to gain 5 – 9 kg over the course of pregnancy.
